# Health risk behaviours among adolescents in Argentina: trends between 2007, 2012 and 2018 national cross-sectional school surveys

**DOI:** 10.1186/s12887-021-02929-0

**Published:** 2021-10-21

**Authors:** Karl Peltzer, Supa Pengpid

**Affiliations:** 1 Department of Psychology, College of Medical and Health Science, Asia University, Taichung, Taiwan; 2 Department of Research Administration and Development, University of Limpopo, Turfloop, South Africa; 3 ASEAN Institute for Health Development, Mahidol University, Salaya, Phutthamonthon, Nakhon Pathom, Thailand

**Keywords:** substance use, physical activity, diet, sexual behaviour, injury, mental health, violence, Argentina

## Abstract

**Background:**

The aim of this study was to assess trends of various health risk behaviours among adolescents across three different surveys in Argentina.

**Methods:**

Data from 115,697 adolescents (mean age:14.6 years, SD=1.2) that participated in three cross-sectional national school surveys in 2007, 2012 and 2018 were analysed. In all, 27 health risk behaviours were assessed through a self-administered questionnaire. Significance of a linear trend was tested by treating study year as categorical variable in logistic regression analyses, adjusted by age group and food insecurity for boys and girls separately

**Results:**

Among both sexes, four health risk behaviours (current cigarette use, passive smoking, trouble from alcohol use, and physically attacked) significantly reduced from 2007 to 2018. Among boys five health risk behaviours (experience of hunger, parental tobacco use, current alcohol use, involvement in physical fighting, and multiple sexual partners), and among girls, inadequate physical activity significantly reduced over time. Among both sexes, the prevalence of four health risk behaviours (overweight/obesity, obesity, leisure-time sedentary behaviour and insufficient fruit intake) significantly increased among both sexes, and among girls ten health risk behaviours (not walking/biking to school, current other tobacco use, bullying victimisation, lifetime drunkenness, having no close friends, suicide plan, suicidal ideation, worry-induced sleep disturbance, loneliness, and ever sexual intercourse) significantly increased over time.

**Conclusion:**

Nine health risk behaviours among boys and five health risk behaviours among girls decreased, and four health risk behaviours among boys and 14 health compromising behaviours among girls increased over a period of 11 years. School health programmes for adolescents should be strengthened in Argentina.

## Background

In Southern Cone States (Uruguay, Paraguay, Chile, Brazil, and Argentina), most death (75.3%) is caused by non-communicable diseases (NCDs), and in Argentina, an upper middle-income country, 78% of death is caused by NCDs [1]. There has been an increase of NCDs and its risk factors in Latin America, including in Argentina [1, 2]. In Southern Cone States, the prevalence of behavioural NCD risk factors in 2016 was 23.5% for adult obesity and 10.2% for adolescent obesity (Argentina 28.3% adult and 14.4% in adolescent obesity), 17.2% and 10.8% for current tobacco smoking among adults and adolescents, respectively (Argentina 21.9% and 20.2% for current tobacco smoking among adults and adolescents, respectively), and 44.3% for adult physical inactivity (Argentina 41.6%) [1]. In a survey among adults in primary care in Central Argentina, the prevalence of inadequate fruit and vegetable intake was 91.8%, physical inactivity 71.5%, dyslipidaemia 43.5%, obesity 35.2%, hazardous alcohol use 28%, and smoking 22.5% [3]. Generally, among adults in Argentina there has an increase of poor diet, physical inactivity, obesity, diabetes, and dyslipidaemia, but a decrease in tobacco use [4]. It is estimated that among young people and adults globally, “alcohol use, dietary behaviours, drug use, mental health, physical activity, sexual behaviours, tobacco use, violence and unintentional injury” are leading causes of morbidity and mortality [5].

In 26 countries in Latin America and the Caribbean 84.5% of adolescents (83.3% in Argentina in 2012) were not physically active [6], and in 12 countries 50% and more adolescents reported sedentary behaviour (50.8 sedentary in Argentina in 2012) [6]. The prevalence of past 12-month physical injury was in 2012-2013, for example 27.1% in Argentina and 29.5% in Uruguay [7]. In a sample of adolescents in Argentina, 6% were traditional bullies [8], and in a national sample of school adolescents in Argentina, the prevalence of past-month bullying victimization was 24.4% [9]. Comparing 13-15 year-old school adolescent from the Argentina 2007 and 2012 Global School-based Student Health Survey (GSHS) and Global Youth Tobacco Survey, found a prevalence of overweight (24.5%) and obesity (4.4%) in 2007 and 28.6% and 5.9%, respectively in 2012, and the prevalence of insufficient vegetable and fruit consumption was 86.0% in 2007 and 82.4% in 2012, physical inactivity 87.3% in 2007 and 83.3% in 2012, sedentary behaviour 49.2% in 2007 and 50.3% in 2012, current smokers 24.5% in 2007 and 19.6% in 2012, and exposure to passive smoking at home 54.7% in 2007 and 44.5% in 2012 [10]. In 21 Latin American and Caribbean Countries, the prevalence of suicidal ideation with planning was 7.5% and 17.5%, among male and female adolescents, respectively, loneliness 10.0% in Argentina in 2012, no close friends 5.3% in Argentina in 2012, and food insecurity 3.8% in Argentina in 2012 [11]. In a study among school adolescents in 25 countries in Latin America and the Caribbean 18.1% reported loneliness and/or having no close friends [12]. The prevalence of current alcohol among 13–15-year-olds in Argentina in 2007 was 51.9% [13], and among adolescents from eight countries in South America and the Caribbean, 18.4% had suicidal ideation, 8.7% anxiety, 15.0% multiple sexual partners and 33.8% school truancy [14]. Among national school adolescent surveys in Bolivia, Costa Rica, Honduras, Peru, and Uruguay, 33.2% had been involved in physical fighting, 37.8% were bullied and 6.7% had not used a condom at last sex [15], while in a large national study among adolescents in Brazil, 30.8% had not used a condom at last sex, and more than 63% had multiple sexual partners (>63%) in Brazil [16]. In terms of protective factors, among adolescents in five Caribbean countries, the prevalence of parental supervision was 38.2%, parental connectedness 32.9% and parental bonding 40.2% [17].

The prevalence of health risk behaviours in adolescents need to be monitored over time at country level to make intervention strategies more appropriate and successful [18, 19]. For example, in a trend study among school-going adolescents in Morocco from 2006 to 2016, “five health risk behaviours (being physically attacked, annual injury, passive smoking, zero days walking or biking to school, and poor hand hygiene after toilet use) significantly declined over time, and inadequate fruit intake and current tobacco use increased over time.” [20]. No trend study among adolescents on various health risk behaviours has been identified in Latin America, including Argentina. Therefore, the aim of this study was assessing trends of 27 health risk behaviours in the 2007, 2012 and 2018 Argentina GSHS. Findings from such a trend study on the epidemiology of health compromising behaviours may help us to understand and design better school health promotion strategies [20].

## Methods

### Sample and procedure

Data from 115,697 adolescents (mean age:14.6 years, SD=1.2) that participated in three cross-sectional national school surveys in 2007, 2012 and 2018 in Argentina were analysed [5]. For the 2007 Argentina GSHS the response rate was 77%, for the 2012 Argentina GSHS 71% and for the 2018 Argentina GSHS 63% [5]. Details of the GSHS and the dataset can be accessed [5]. Briefly, “using a two-stage cluster sampling strategy (schools were selected by probability to size sampling and random selection of classrooms with students 13 to 17 years of age), nationally representative samples of middle school students were produced.” [5] “All students who attended a selected class were eligible to participate, regardless of their age, and completed a self-administered questionnaire in their language under the supervision of trained external survey administrators.“ [5] The study was granted ethics approval by a national ethics committee and written informed consent was obtained from the participants or their guardians before the survey [5].

#### Measures

All health risk behaviours of the GSHS measure [5] that were administered in the 2007, 2012 and 2018 Argentina GSHS were included in this study (see Table 1). This included body weight and dietary behaviour (overweight or obesity, obesity, food insecurity or hunger, and vegetable and fruit intake), leisure-time sedentary behaviour, walking or biking to school, and physical activity, substance use (parental tobacco use, current cigarette use, current other tobacco use, passive smoking, current alcohol use, drunkenness, trouble as a result of drinking alcohol), injury and violence (bullied, attacked, and in a physical fight), psychological health (having friends, loneliness, worry-induced sleep problems, suicide plan, and suicidal ideation), and sexual behaviour (ever sexual intercourse, multiple sexual partners, and non-condom use). In addition, protective measures included peer support, school attendance, and parental support. The intake of “less than two or more servings of fruits in a day” and “less than three or more servings of vegetables a day” were classified as inadequate [21]. “Inadequate physical activity was defined as not daily at least 60 minutes of moderate to vigorous-intensity physical activity.” [22]. “Leisure-time sedentary behaviour was defined as spending three or more hours per day sitting.” [23].

**Table 1:** Description of study variables.

Variables	Question	Response options (coding scheme)
Age	“How old are you?”	“11 years old or younger to 16 or 18 years old or older”
Sex	“What is your sex?”	“Male, Female”
Grade	“In what grade/class/standard are you?”	
Food insecurity	“During the past 30 days, how often did you go hungry because there was not enough food in your home?"	“1= never to 5= always” “(coded 1=0, 2-3=2 and 4–5=1)”
**Body weight and dietary behaviour**		
Height	“How tall are you without your shoes on?”	
Weight	“How much do you weigh without your shoes on?”	
Fruits	“During the past 30 days, how many times per day did you usually (past 7 days how many times did you) eat fruit such as apples, bananas, or mandarins?”	“1=I did not eat fruit during the past 30 days/7 days to 7=5 or more/4 or more times per day” “(coded <2 times/day)”
Vegetables	“During the past 30 days/7 days, how many times per day did you usually (past 7 days how many times did you) eat vegetables, such as lettuce, tomatoes, carrots, or pumpkin?”	“I did not eat vegetables during the past 30 days to 7=5 or more/4 or more times per day” “(coded <3 times/day”
Hunger	“During the past 30 days, how often did you go hungry because there was not enough food in your home?"	“1= never to 5= always” “(coded 1–3=0 and 4–5=1)”
**Physical activity and sedentary behaviour**		
“Physical activity”	“Physical activity is any activity that increases your heart rate and makes you get out of breath some of the time. Physical activity can be done in sports, playing with friends, or walking to school. Some examples of physical activity are running, fast walking, biking, dancing, football, swimming, or skating.” “During the past 7 days, on how many days were you physically active for a total of at least 60 minutes per day?”	“0=0 days to 7=7 days” “(coded 0-6=0 and 7=1)”
“Leisure-time sedentary behaviour”	“How much time do you spend during a typical or usual day sitting and watching television, playing computer games, talking with friends, or doing other sitting activities, such as using the computer or cell phone?”	“1=less than 1 hour per day; 2=1-2 hrs/day; 3=3-4 hrs/day; 4=5-6 hrs/day; 5=7-8 hrs/day and 6=8 or more hours per day”
“Walking or biking to school”	“During the past 7 days, on how many days did you walk or ride a bicycle to or from school?”	“1=0 days to 8=7 days” “(coded 1=1 and 2–8=0)”
**Substance use**		
“Parental tobacco use”	“Which of your parents or guardians use any form of tobacco?”	“Neither, My father or male guardian, My mother or female guardian, Both” “(coded 0=neither and 1=either)”
“Current smoking cigarettes”	“During the past 30 days, on how many days did you smoke cigarettes?”	“1=0 days to 7=All 30 days” “(coded 1=0 and 2-7=1)”
“Current other tobacco use”	“During the past 30 days, on how many days did you use any tobacco products other than cigarettes, such as pipes, narguile, or smokeless tobacco?”	“1=0 days to 7=All 30 days” “(coded 1=0 and 2-7=1)”
“Passive smoking”	“During the past 7 days, on how many days have people smoked in your presence?”	“1=0 days to 5=all 7 days” “(coded 1=0 and 2-5=1)”
“Current alcohol use”	“During the past 30 days, on how many days did you have at least one drink containing alcohol?”	“1=0 days to 7=All 30 days” “(coded 1=0 and 2-7=1)”
“Drunk”	“During your life, how many times did you drink so much alcohol that you were really drunk?”	“1=0 times to 4=10 or more times” “(coded 1=2=4 and 0=1)”
“Trouble from alcohol use”	“During your life, how many times have you got into trouble with your family or friends, missed school, or got into fights, as a result of drinking alcohol?”	“1=0 times to 4=10 or more times” “(coded 1=2=4 and 0=1)”
**Injury and violence**		
“Injury”	“During the past 12 months, how many times were you seriously injured?”	“1=0 times to 8=12 or more times” “(coded 1=0 and 2–8=1)”
“Bullied”	“Bullying occurs when one or more students or someone else about your age teases, threatens, ignores, spreads rumors about, hits, shoves, or hurts another person over and over again. It is not bullying when two people of about the same strength or power argue or fight or tease each other in a friendly way?” GSHS 2007 and 2012 “During the past 30 days, on how many days were you bullied?” GSHS 2018 “During the past 12 months, have you ever been bullied on school property?” “During the past 12 months, have you ever been bullied when you were not on school property?”	“1=0 days to 7=All 30 days” “(coded 1=0 and 2–7=1)” “Yes, No” “Yes, No” (coded any of the two=1)
“In a physical fight”	“During the past 12 months, how many times were you in a physical fight?”	“1=0 times to 8=12 or more times (coded 1=0 and 2–8=1)”
“Physically attacked”	“During the past 12 months, how many times were you physically attacked?”	“1=0 times to 8=12 or more times (coded 1=0 and 2–8=1)”
**Poor mental health**		
“No close friends”	“How many close friends do you have?”	“1 = 0 to 4 = 3 or more (coded 1+=0, 0=1)”
“Loneliness”	“During the past 12 months, how often have you felt lonely?”	“1=never to 5=always (coded 1–3=0 and 4–5=1)”
“Worry-induced sleep disturbance”	“During the past 12 months, how often have you been so worried about something that you could not sleep at night?”	“1=never to 5=always (coded 1–3=0 and 4–5=1)”
“Suicidal ideation”	“During the past 12 months, did you ever seriously consider attempting suicide?”	“Yes, No”
“Suicide plan”	“During the past 12 months, did you make a plan about how you would attempt suicide?”	“Yes, No”
**Sexual behaviour**		
“Ever had sex”	“Have you ever had sexual intercourse?”	“Yes, No”
“Multiple sexual partners”	“During your life, with how many people have you had sexual intercourse?”	“1=never had sex to 7=6 or more people (coded 1=3-7 and 0=1-2)”
“Non-condom use”	“The last time you had sexual intercourse, did you or your partner use a condom, forro, preservativo, or profilactico?”	“Never had sex/Yes/No (coded 1=No and 0=never had sex/yes)”
**Protective factors**		
“School attendance”	“During the past 30 days, on how many days did you miss classes or school without permission?”	“1=0 days to 5=10 or more days (coded 1=1 and 2-5=0)”
“Peer support”	“During the past 30 days, how often were most of the students in your school kind and helpful?”	“1=never to 5=always (coded 1–3=0 and 4–5=1)”
“Parental supervision”	“During the past 30 days, how often did your parents or guardians check to see if your homework was done?”	“1=never to 5=always (coded 1–3=0 and 4–5=1)”
“Parental connectedness”	“During the past 30 days, how often did your parents or guardians understand your problems and worries?”	“1=never to 5=always (coded 1–3=0 and 4–5=1)”
“Parental bonding”	“During the past 30 days, how often did your parents or guardians really know what you were doing with your free time?”	“1=never to 5=always (coded 1–3=0 and 4–5=1)”

#### Data analysis

Cross-sectional national datasets from three Argentina GSHS in 2007, 2012 and 2018 were merged, and weighted for probability selected and non-response. Chi-square tests were utilized for analysing differences in proportions, and descriptive health risk behaviour information was reported as percentages for each study year. The significance of linear trends was analysed by using study year as categorical variable in logistic regression analyses, adjusted by age group and food insecurity for boys and girls separately. Taylor linearization methods were used in statistical analyses accounting for sample weight and multi-stage sampling. Missing data were excluded from the analyses, and *p*<0.05 was accepted as significant. All statistical analyses were done using STATA software version 15.0 (Stata Corporation, College Station, Texas, USA).

## Results

### Sample characteristics

The total sample included 115,697 adolescents (Mean age:14.6 years, SD=1.2), and 51.9% were females. Compared to the first two surveys, the students attending ’2nd year/11th grade polymodal or 4th year of high school’ or ’3rd year/12nd grade polymodal or 5th year of high school’ were included in the third survey (*p*<0.001) (see Table 2).

**Table 2:** Characteristics of adolescent students for 2007, 2012 and 2018 surveys in Argentina

Variable	2007 (*N*=1,980)	2012 (*N*=56,736)	2018 (*N*=56,981)
	N (%)	N (%)	N (%)
Sex			
Male	957 (48.0)	26728 (48.3)	27083 (48.0)
Female	994 (52.0)	29248 (51.7)	29362 (52.0)
Missing	29	760	536
Age in years			
13 years or younger	370 (18.7)	11412 (26.0)	10767 (21.0)
14	600 (27.0)	16354 (29.5)	12946 (25.0)
15	576 (29.6)	15448 (25.4)	12812 (22.0)
16 years or older	400 (24.8)	12812 (19.2)	20348 (31.9)
Missing	34	468	108
Grade			
A	732 (38.4)	28576 (37.2)	8581 (14.7)
B	880 (32.0)	19944 (33.5)	11913 (26.9)
C	337 (29.7)	16574 (29.3)	12448 (23.3)
D-E	0	0	22978 (35.1)
Missing	31	1642	1061
Food insecurity (as proxy for socioeconomic status)			
None	1321 (67.1)	35222 (64.4)	38257 (68.4)
Moderate	588 (29.8)	18680 (31.7)	17162 (29.6)
Severe	58 (3.1)	2140 (3.9)	1063 (2.0)
Missing	13	694	499

A=’8th grade of primary school/polimodal or 1st year of high school’; B=’9th grade of primary school/polimodal or 2nd year of high school’; C=’1st year/10th grade polimodal or 3rd year of high school’; D=’2nd year/11th grade polimodal or 4th year of high school’; E=’3rd year/12nd grade polimodal or 5th year of high school.’

### Outcome variables

#### Body weight and dietary behaviour

The prevalence of overweight or obesity was 21.5% among boys and 12.4% among girls in 2007, which significantly increased to 35.2% among boys and 25.9% among girls in 2018. Similarly, the proportion of obesity increased among both boys and girls from 2007 (3.0% and 1.9%, respectively) to 2018 (9.7% and 5.2%, respectively). The prevalence of inadequate fruit consumption increased from 70.6% to 79.6% among boys and from 65.4% to 78.6% among girls from 2007 to 2018, while the prevalence of inadequate vegetable intake did not significantly change among both boys and girls over time. Experiencing hunger was low and decreased significantly in boys from 4.3% in 2007 to 2.3% in 2018 but did not change among girls over time.

#### Physical activity and sedentary behaviour

Both male and female adolescents reported a high prevalence of physical inactivity (boys: 82.7% and girls: 92.2%), which decreased significantly among girls (87.1%) and remained unchanged among boys (79.6%) over time. Leisure-time sedentary behaviour significantly increased among both boys from 44.7% in 2007 to 52.8% in 2018 and girls from 51.0% in 2007 to 57.7% in 2018. Not walking/biking to school significantly increased from 26.2% in 2007 to 34.0% in 2018 among girls but remained unchanged among boys.

#### Substance use

Among both boys and girls, parental tobacco use decreased over time from 38.4% to 33.8% among boys and 38.6% to 35.2% among girls. The prevalence of current cigarette use, significantly decreased from 24.3% in 2007 to 17.2% in 2018 among boys and from 26.9% in 2007 to 20.6% in 2018 among girls. However, among girls, current other tobacco use, almost doubled from 4.3% in 2007 to 8.0% in 2018, while among boys, current other tobacco use remained unchanged. The proportion of passive smoking reduced significantly among both boys and girls over time. Current alcohol use, significantly declined among boys but not among girls over time. Trouble from alcohol use decreased significantly among both boys and girls from 2007 to 2018, while lifetime drunkenness significantly increased among girls from 31.2% in 2007 to 38.8% in 2018 and remained unchanged among boys.

#### Violence and injury

The proportion of injury did not change significantly among boys and girls from 2007 to 2018. Bullying victimisation increased in both boys and girls, but only in girls significantly from 23.6% in 2007 to 34.9% in 2018. Being physical assaulted reduced in both sexes over time from 30.2% to 19.3% among boys and from 19.2% to 15.9% among girls and participation in physical fighting decreased significantly among boys from 43.8% to 33.3% and reduced among girls from 19.6% to 16.5% but not significantly.

#### Psychological health

All five indicators (“having no close friends, worry-induced sleep disturbance, loneliness, suicidal ideation and suicide plan”) significantly increased among girls but not among boys over time.

#### Sexual behaviour

Ever having had sexual intercourse significantly increased among girls from 24.4% in 2007 to 36.4% in 2018 but remained unchanged among boys. The prevalence of having multiple sexual partners significantly reduced from 34.8% to 28.4% among boys but not among girls (from 13.6% to 16.9%). The proportion of non-condom use at last sex remained unchanged among both boys and girls over time.

#### Protective indicators

School attendance in the past 30 days significantly increased among both boys and girls from 2007 to 2018. Peer support significantly decreased among girls but not boys over time. Parental supervision and connectedness decreased significantly among both boys and girls from 2007 to 2018, while parental bonding did not change over time (see Tables 3 and 4).

**Table 3:** Health risk behaviours among male adolescents in 2007, 2012 and 2018 in Argentina

***Outcome variable***	2007	2012	2018	Difference^a^	p-for trend^b^
	%	%	%		
***Body weight and dietary behaviour***					
Overweight/obesity	21.5	35.0	35.2	+13.7	<0.001
Obesity	3.0	8.9	9.7	+6.7	<0.001
Fruits <2 times/day	70.6	64.6	79.6	+9.0	<0.001
Vegetable <3 times/day	92.3	87.6	89.9	-2.4	0.076
Went hungry (mostly/always)	4.3	4.6	2.3	-2.0	0.013
***Physical activity and sedentary behaviour***					
Physical inactivity	82.7	78.1	79.6	-3.1	0.220
Leisure-time sedentary behaviour	44.7	46.9	52.8	+8.1	0.003
0 days walk or bike to school	27.0	31.3	30.0	+3.0	0.485
***Substance use***					
Parental tobacco use	38.4	37.6	33.8	-4.6	0.036
Current cigarette use	24.3	19.2	17.2	-7.1	<0.001
Current other tobacco use	8.3	8.8	8.2	-0.1	0.965
Passive smoking	77.9	71.0	64.3	-13.6	<0.001
Current alcohol use	61.5	52.0	52.4	-9.1	0.008
Lifetime drunk	38.7	32.8	35.7	-3.0	0.299
Trouble from alcohol use	25.2	22.3	12.6	-12.6	<0.001
***Injury and violence***					
Any serious injury	42.2	42.4	38.4	-3.8	0.157
Bullied	26.6	24.3	29.7	+3.1	0.094
In physical fight	43.8	44.7	33.3	-10.5	<0.001
Physically attacked	30.2	30.4	19.3	-10.9	<0.001
***Poor mental health***					
Having no close friends	4.5	6.6	5.9	+1.4	0.073
Loneliness	7.5	5.8	10.1	+2.6	0.053
Worry-induced sleep disturbance	7.6	5.7	8.1	+0.5	0.362
Suicidal ideation	14.0	11.7	13.8	-0.2	0.773
Suicide plan	11.2	11.9	11.0	-0.2	0.960
**Sexual behaviour**					
Ever sex	44.4	48.2	48.2	+3.8	0.298
Multiple sexual partners	34.8	25.9	28.4	-6.4	0.036
Non-condom use	10.2	8.9	7.7	-2.5	0.055
***Protective factors***					
School attendance	60.0	65.9	68.3	+8.3	0.002
Peer support (mostly/always)	50.1	47.1	46.8	-3.3	0.316
Parents/guardians supervision (mostly/always)	37.7	32.4	30.4	-7.3	0.005
Parents/guardians connectedness (mostly/always)	52.6	46.8	42.9	-9.7	<0.001
Parents/guardians bonding (mostly/always)	53.9	49.6	53.2	-0.7	0.837

^a^Difference between 2007 and 2018; ^b^Adjusted for age and socioeconomic status (food insecurity)

**Table 4:** Health risk behaviours among female adolescents in 2007, 2012 and 2018 in Argentina

***Outcome variable***	2007	2012	2018	Difference^a^	p-for trend^b^
	%	%	%		
***Body weight and dietary behaviour***					
Overweight/obesity	12.4	21.0	25.9	+13.5	<0.001
Obesity	1.9	3.4	5.2	+3.3	<0.001
Fruits <2 times/day	65.4	59.1	78.6	+13.2	<0.001
Vegetable <3 times/day	90.8	86.4	89.2	-1.6	0.427
Went hungry (mostly/always)	1.8	3.1	1.7	-0.1	0.483
***Physical activity and sedentary behaviour***					
Physical inactivity	92.2	87.8	87.1	-5.1	<0.001
Leisure-time sedentary behaviour	51.0	52.8	57.7	+6.7	0.036
0 days walk or bike to school	26.2	32.1	34.0	+7.8	0.046
***Substance use***					
Parental tobacco use	38.6	35.8	35.2	-3.4	0.114
Current cigarette use	26.9	21.3	20.6	-6.3	0.005
Current other tobacco use	4.3	5.8	8.0	+3.7	0.006
Passive smoking	78.5	76.7	68.4	-9.9	<0.001
Current alcohol use	53.2	51.3	55.7	+2.5	0.290
Lifetime drunk	31.2	29.5	38.8	+7.6	<0.001
Trouble from alcohol use	20.8	21.8	13.3	-8.5	<0.001
***Injury and violence***					
Any serious injury	26.2	25.3	28.4	+2.2	0.171
Bullied	23.6	23.5	34.9	+11.3	<0.001
In physical fight	19.6	24.1	16.5	-3.1	0.075
Physically attacked	19.2	19.8	15.9	-3.3	0.042
***Poor mental health***					
Having no close friends	4.0	4.7	5.3	+1.3	0.043
Loneliness	12.8	13.1	23.1	+10.3	<0.001
Worry-induced sleep disturbance	13.7	11.9	18.0	+4.3	<0.001
Suicidal ideation	19.8	22.2	28.6	+8.8	<0.001
Suicide plan	15.8	19.6	22.6	+6.8	<0.001
**Sexual behaviour**					
Ever sex	24.4	35.2	36.4	+12.0	<0.001
Multiple sexual partners	13.6	14.9	16.9	+3.3	0.210
Non-condom use	7.0	7.8	8.4	+1.4	0.532
***Protective factors***					
School attendance	65.7	69.3	72.3	+6.6	0.016
Peer support (mostly/always)	57.8	53.8	44.5	-13.3	<0.001
Parents/guardians supervision (mostly/always)	37.0	28.6	26.6	-10.4	<0.001
Parents/guardians connectedness (mostly/always)	54.9	49.7	40.0	-14.9	<0.001
Parents/guardians bonding (mostly/always)	59.2	58.0	56.9	+0.7	0.116

^a^Difference between 2007 and 2018; ^b^Adjusted for age and socioeconomic status (food insecurity)

## Discussion

Results show for the first time that across three GSHS in 2007, 2012 and 2018 in Argentina, among both sexes a significant decrease in the prevalence of current cigarette use, passive smoking, trouble from alcohol use, and physically attacked, and among boys, experience of hunger, parental tobacco use, current alcohol use, involvement in physical fighting, and multiple sexual partners, and among girls, inadequate physical inactivity. However, overweight/obesity, obesity, leisure-time sedentary behaviour and insufficient fruit intake significantly increased among both boys and girls, and among girls not walking/biking to school, current other tobacco use, bullying victimisation, lifetime drunkenness, having no close friends, loneliness, worry-induced sleep disturbance, suicidal ideation, suicide plan, and ever sexual intercourse significantly increased over time.

The significant reduction in current cigarette use, and passive smoking, also found in the Argentina Global Youth Tobacco Survey [10], may be attributed to the introduction of the smoke-free law in Argentina in 2011, including a “total ban on smoking in public settings, prohibition of advertising and promotional activities regarding tobacco use, and enforcing manufacturers to include messages warning of the harmful effects of cigarette smoking on health.” [24, 25]. However, of concern is that the prevalence of other tobacco use, significantly increased among girls from 2007 to 2018. Current alcohol decreased among boys, trouble from alcohol use decreased among both boys and girls, and lifetime drunkenness increased among girls. Current alcohol use is high in both sexes (52% among boys and 56% among girls) and is among adolescents often associated with negative health outcomes, such as interpersonal violence [13]. Public health interventions may be indicated to reduce alcohol use among adolescents in Argentina [13]. Although some national policies and interventions are in place for alcohol use in Argentina, such as legal minimum age for on or off premise sales of alcoholic beverages (18 years), and legally binding regulations on alcohol advertising, health warning labels on alcohol advertisements, there is no written national alcohol policy, no legally binding regulations on alcohol sponsorship, and no restrictions for on-/off-premises sales of alcoholic beverages [26].

Overweight/obesity and obesity, sedentary behaviour, not walking/biking to school (particularly among girls), and inadequate fruit consumption increased from 2007 to 2018, which may be attributed to a nutritional transition (to increased intake of processed foods, sugar-sweetened soft drinks or juices, and reduction of total fruit consumption) in Argentina [27]. The increase in sedentary behaviour may be attributed to an increased internet and mobile devices use among adolescents in Argentina [28]. Among girls, the prevalence of physical inactivity decreased, and was among both boys and girls like global rates (85%) [29]. However, the prevalence of sedentary behaviour was much higher than global estimates among adolescents (30%) [29]. It is possible that not walking/biking to school increased among girls because of lower parental supervision and peer support [30], as found in this study. The extent of food insecurity was below 2.4% in 2018, decreased among boys and remained unchanged among girls. The federal in-school feeding programme may have had a positive impact to reduce food insecurity [31].

In line with some previous trend studies [18, 32–34], this study showed that physically assaulted and involvement in physical fighting declined over time. Perhaps, one factor contributing to the decline in interpersonal violence, is the decline of alcohol use among boys in this study [13]. In addition, we found an increase in school attendance in our study, which may also have contributed to the decline of interpersonal violence. Compared to boys, bullying victimisation increased significantly among girls. It is possible that the decline in peer support among girls in this study contributed to an increase in being bullied among girls. The prevalence of bullying victimization in this study was higher in girls than in boys, which has also been reported in a different study in Argentina, Uruguay, and Brazil [35]. The overall participation in physical fighting (30.0%) and bullying victimisation (27.3%) in this study were lower than among adolescents from Bolivia, Costa Rica, Honduras, Peru, and Uruguay (33.2% had been involved in physical fighting 37.8% were bullied) [15].

Regarding sexual behaviour, among boys, sexual risk behaviour (multiple sexual partners and non-condom use) decreased over time, and girls ever sexual intercourse increased over time. The since 2006 a national programme on comprehensive sexual education curriculum has been integrated across school levels [36], which may have contributed to low sexual risk behaviour. Compared to studies among adolescents in South America, the overall prevalence of non-condom use (8.3% overall and 22.9% among sexually active) was similar to Bolivia, Costa Rica, Honduras, Peru, and Uruguay (6.7%) [15] and lower than in Brazil (30.8% among sexually active) [16], and the overall prevalence of multiple sexual partners (21.9% overall and 53.2% among sexually active) in this study was lower than in Brazil (>63% among sexually active) in Brazil [16].

Among boys, all mental health indicators remained unchanged, while among girls all five mental health indicators (suicide plan, loneliness, suicidal ideation, no close friends, and worry-induced sleep disturbance) significantly increased from 2007 to 2018. In response to this a gender-responsive health for suicide prevention programme through the establishment of school-based health advisory services has recently been implemented and is being roll-out in Argentina [37]. The overall prevalence of suicidal ideation (18.4%) in this study was similar to the study among adolescents in five Latin American countries (19.5%) [15].

Regarding protective aspects, school attendance increased among both boys and girls, while peer support significantly decreased among girls but not among boys. Parental supervision and connectedness significantly decreased among both boys and girls over time in this study. This decline in parental support may be related to recent changes in family transformations in Argentina, including “increases in the age at marriage, marital dissolution, nonmarital births, and cohabitation, and with women increasingly contributing to the economic support of their families.” [38]. It is possible that the decline of parental supervision and connectedness contributed to poorer mental health, in particular among girls in this study. Overall, the prevalence of parental support (parental supervision 31.2%, parental connectedness 47.1% and parental bonding 54.9%) in this study was lower in terms parental supervision (38.2%) but higher regarding parental connectedness (32.9%) and parental bonding (40.2%) than among adolescents in five Caribbean countries [17].

Study findings highlight a wide range of health risk behaviours that can be targeted in school health promotion activities among in Argentina. Comprehensive Protection of the Rights of Boys, Girls and Adolescents, PROSANE is developed as an “Integrated Care Policy for children and adolescents”. ‘PROSANE is part of the Primary Health Care strategy strengthening the link between the school and the health centre makes it possible to identify issues that require promotional actions in schools. PROSANE promotes and develops health promotion actions in conjunction with teachers, managers, and families, promoting learning and integral human development, improving the quality of life and the collective well-being of children and adolescents and other members of the community [39].

### Study limitations

“Secondary education net-enrolment ratio” was 85.9% in Argentina in 2012 and 90.7% in 2018 [40], which implies that some adolescents not attending school were not included in this study in Argentina. Some GSHS study variables, such as oral and hand hygiene, soft drink and fast-food consumption, were not included in this paper, since they were only assessed in one or two waves of the Argentina GSHS. The study design was cross-sectional, which precludes from causal inferences. The GSHS collected anonymously data by self-report that could have contributed to some bias but may nevertheless have reported valid data, in particular on sensitive issues [41].

## Conclusions

Nine health risk behaviours (current cigarette use, passive smoking, trouble from alcohol use, physically attacked, experience of hunger, parental tobacco use, current alcohol use, and involvement in physical fighting) among boys and five health risk behaviours (current cigarette use, passive smoking, trouble from alcohol use, physically attacked, and inadequate physical inactivity) among girls decreased, and four health risk behaviours (overweight/obesity, obesity, leisure-time sedentary behaviour and insufficient fruit intake) among boys and 14 health compromising behaviours (overweight/obesity, obesity, leisure-time sedentary behaviour and insufficient fruit intake, not walking/biking to school, current other tobacco use, bullying victimisation, lifetime drunkenness, having no close friends, loneliness, worry-induced sleep disturbance, suicidal ideation, suicide plan, and ever sexual intercourse) among girls increased over a period of 11 years. School health programmes for adolescents should be strengthened in Argentina.

## Data Availability

The datasets generated during and/or analysed during the current study are available in the World Health Organization NCD Microdata Repository, https://extranet.who.int/ncdsmicrodata/index.php/catalog/ Public access to this database is open.

## Declarations

All methods were carried out in accordance with relevant guidelines and regulations and have been performed in accordance with the Declaration of Helsinki.
